# Reference values for resting and post exercise hemodynamic parameters in a 6–18 year old population

**DOI:** 10.1038/s41597-020-0368-z

**Published:** 2020-01-21

**Authors:** Katalin Havasi, Zoltán Maróti, Andrea Jakab, István Raskó, Tibor Kalmár, Csaba Bereczki

**Affiliations:** 1 Csongrád County Health Care Center Hódmezővásárhely-Makó, Csongrád, Hungary; 2 Department of Pediatrics, University of Szeged Albert Szent-Györgyi Medical Center Faculty of Medicine, Szeged, Hungary; 3 Inst. of Genetics, Biological Research Center, Szeged, Hungary

**Keywords:** Risk factors, Health care

## Abstract

This database is the first large dataset of haemodynamic changes of normal-weight pupils during a field exercise test. Here, we present a dataset for anthropometric and hemodynamic parameters measured both during relaxation and after exercise containing 1,173,342 data segments from 65,345 acquisition points of 10,894 normal weight subjects, covering an age range of 6–18 years collected in a course of 12 years. Data acquisition was carried out under standardised measuring conditions and specifications. Hemodynamic parameters were measured in the normal-weight population with a new and simple Fit-Test which could facilitate new projects worldwide to study and compare cardiovascular fitness.

## Abstract (summary)

**Table:** 

Measurement(s)	anthropometric measurement • heart rate • blood pressure
Technology Type(s)	anthropometric analysis • Cardiac Monitoring
Factor Type(s)	age
Sample Characteristic - Organism	Homo sapiens

Machine-accessible metadata file describing the reported data: 10.6084/m9.figshare.11417481

## Background & Summary

Despite advances in diagnosis and treatment over the past 30 years, the disability-adjusted life years (DALY) attributable to hypertension have increased worldwide by 40% since 1990^1–5^. Hypertension in children and adolescents is becoming a major concern, not only because of its rising prevalence but because almost half of the adults with hypertension had elevated blood pressure values during their childhood^4,6,7^.

As already established, elevated blood pressure in childhood correlates with carotid intima-media thickness, atherosclerosis, left ventricular hypertrophy, and kidney failure in adulthood^8,9^. Consequently, early diagnosis and control of hypertension in childhood are likely to have an important effect on long-term outcomes of hypertension-related cardiovascular complications^10^.

Underdiagnosis of hypertension in children and adolescents is the consequence of using only (casual) BP values while they are resting in an office environment. When defining high BP, especially in younger age groups, various influences limit the reliability of the in-office BP measurements^11–14^. On the other hand out-of-office BP might be a more reliable parameter than casual BP, which has a strong association with cardiovascular disease outcome^15^. Furthermore, exercise BP and cardiopulmonary fitness has a robust, inverse, and independent association with cardiovascular and overall mortality risk^16–18^.

Consequently, the predictive power for cardiovascular disease of an exaggerated BP response during exercise suggested being superior to resting BP not only in adult populations but in childhood and adolescents as well^17^. Cardiopulmonary exercise testing is an essential tool to assess cardiorespiratory fitness (CRF) in children, since exercise hypertension has been suggested to predict future resting hypertension^13,19^. While these observations highlight the potential clinical utility of exercise BP measurements for diagnostic and prognostic purposes, they have yet to be widely adopted into clinical (and non-clinical) practice given the limitations, such as the lack of standardized methodology and limited empirical evidence across a wide range of populations. Our knowledge of the CRF and its relevance, especially in relation to the whole population, caused by the physical exercise of children and adolescents, is incomplete, with very few publications^19–22^.

Currently, only a small number of screening methods enable simple determination of cardiorespiratory fitness, particularly in children. Most of these methods need a special environment and special conditions^22,23^.

The Distance Running Test (DRT) is a good alternative to ergometer exercise measuring haemodynamics variables during exercise in childhood. Although the accuracy of the CRF definition outside the laboratory is necessarily lower than that of the laboratory measurements, but since haemodynamic parameters significantly correlate with the maximal aerobic speed (MAS) during the ergometer test, and MAS could have been predicted from average speed during DRT, it gives us a simple, standardisable option to test CRF by measures of haemodynamic parameters, pulse and blood pressure.

**Fig. 1:**
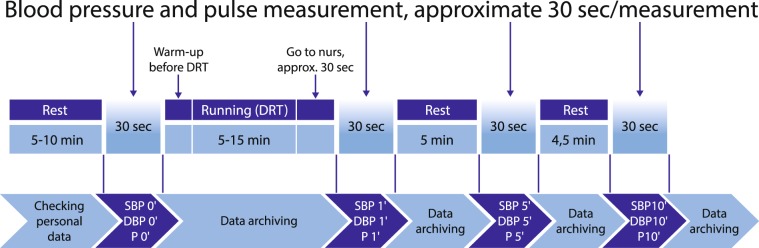
Measurement/Fit-test Protocol. The duration of the whole experiment was approximately 40–45 minutes. Four pulse (P), systolic (SBP) and diastolic blood pressure (DBP) measurements were collected per subject during a single Fit-test.

Here, we suggest a field test (Fig. 1). Specifically, a 1000 meter DRT for 1–4 classes (age between 6–10 years) and a 2000 meter DRT (for 5–13 classes age between 10–18 years) which are the best predictors of cardiorespiratory fitness (CRF) according to latest studies^24–26^. It is an alternative to laboratory stress tests for screening CRF and calculating the age and gender-specific percentiles associated with it. Such a test, in addition to establishing the reference values also provides an opportunity to gain new insight into the relationship between later manifestations of illness and juvenile burden response.

In order to achieve this we tested more than 14,000 6–18 years old pupils for 12 years containing repeated measures on the same individuals. Here we provide the registered anthropometric and cardio-metric data (pre- and post-test blood pressure and pulse values) collected during the Fit-test for those with a calculated BMI within the normal range, according to the published WHO guideline^26^.

## Methods

### Participant characteristics

Ethical License: This research was conducted with the permission of the Research Ethics Committee of the University of Szeged.

This investigation was conducted as a prospective, multicenter study in 3 Hungarian cities (Hódmezővásárhely, Mártély and Mindszent), in southern Hungary between 2007 and 2018. In order to exclude the possibility of error in the selection of the examined subpopulation, we aimed to accomplish a comprehensive survey of the students of Hódmezővásárhely, which is the most populated of the three cities. Population: 43,700 (2018) mainly Caucasian type, (http://www.ksh.hu/nepszamlalas/tablak_teruleti_06) self-declaration basis, CSO census: in the region, 1.9% Roma population, other races do not occur in larger numbers). Inward and outward migration was negligible in the period under review.

The involvement in the anthropological measurements in the school is compulsory for everyone. Participation in physical education is also mandatory for all students except for those who are excluded from physical education. The additional BP parameter measurements before and after the running-test was optional, but no one opted out, all participants gave consent.

### Measurement protocol/data collection

The survey was performed at the beginning and at the end of the school year, except for those who were excluded from physical exercise and data was recorded in an IT system. The measurements were obtained during regular Physical Education (PE) classes for children between 8 and 14.

#### Biometric Data

Anthropometric data including weight, height measured by trained data collectors^26^. In the school-health offices, certified, calibrated, non stretchable, wall-mounted stadiometer height measuring equipment was used for measuring pupils‘ heights. Hair styles and hair accessories were removed or undone. The participant were asked to stand in socks against the stadiometer, with heels together, legs straight, arms at sides, and shoulders relaxed, looking straight and were measured standing with heels, buttocks, shoulders and head touching a flat upright surface of the stadiometer. The perpendicular headpiece brought down to touch the crown of the head had enough pressure to compress the hair. The measurer’s eyes were parallel with the headpiece in order to read the measurement to the nearest cm.

A certified, calibrated electronic scale with a tare capability was used to measure the weight of the participants. They were weighed wearing lightweight underwear (shoes, hats or bulky items such as coats/jackets and sweaters were removed) standing, without assistance, in the middle of the scale platform. The weight to the nearest 0.1 kg was noted and was rounded to whole kg. Every year an external company calibrated all the devices used for measurements.

In the hours before the measurements, the students were not exposed to significant physical exertion. The students from the nearby schools came to the survey on foot and from remote schools by school buses. The anthroplogical measurements carried out before the running test by school doctors/nurses in the office indoor with calibrated instruments. Blood pressure and pulse values were measured with a validated, automatic OMRON blood pressure monitor, in accordance with the daily practice of school screening and the Hungarian Hypertension Society (MHT) protocol^27^. On arrival they sat on chairs and benches for about 5–10 minutes. From there the nurse called for a measurement. The whole class was surveyed at the same time, with an average of 20–30 students. A nurse group (of 10 to15 nurses) performed blood pressure and heart rate measurements on the tables alongside the track. Thus, an experienced nurse usually measured the blood pressure of two students in turn. The students’ blood pressure and heart rate were measured on a chair with a back rest, with her/his arm placed comfortably, at heart level (SBP 0′, DBP 0′, Pulse 0′).

The running test was concluded as follows: the whole class (except children excluded from excersice) was tested outdoors. After the initial measurement, before the survey, the PE teacher performed warm-up exercises with the students. During the test, under the supervision of physical educators, everyone had to run 1000 meters/0.62 miles (1–4 classes age between 6–10) or 2000 meters/1.24 miles (5–13 classes age between 10–18) as fast as possible on the same 400 meter (0.24 miles) long, flat, oval outdoor track. The PE teacher measured the run time with a manual stopwatch and recorded the result of the run. After completing the distance, the student immediately went to one of the nurses sitting at the tables next to the course, who measured her/his blood pressure (SBP 1′, DBP 1′, Pulse 1′) and transferred her/him to another chair and informed her/him of the time of the next measurment. The nurse called the student she had previously measured for both the 5-minute and 10-minute measurements (SBP 5′, DBP 5′, Pulse 5′ and SBP 10′, DBP 10′, Pulse 10′), and recorded the data immediately after each measurement. All BP measurements were carried out once in each time point.

Students were excluded from Physical Education: with severe cardiopulmonary, pulmonary or musculoskeletal disorders; acute fever patients, acute exacerbation of asthma, very high BP values and/or complaints were exempt from the exercise. Students with high blood pressure but no complaints and those who were controlled by medication for asthma bronchiale, hypertension, diabetes mellitus, cystic fibrosis, and mild scoliosis also participated.

On average 77.27% of the participants completed the running test, while the rest of them were either excluded from PE class or were absent from the test due to missing class on the day for any other reason.

### De-identification

In the process of creating the dataset, all identifiable personal information has been removed. Each individual thus has only an ID that links her/his measurements at different dates in the database.

### Data screening

To ensure high-quality dataset we performed a data integrity screening for the measured parameters. Since the actual height of an individual could vary (~1–2 cm) even in a course of a day (as physical activity alters spinal length) and the measured height also depends on how much the subject draws herself/himself up at the measurement time. We would expect a natural variation of height data even when the height of the individual is the same. Furthermore the measured height was rounded to the nearest integer, so it could also cause 1 cm difference in height without having significant difference measured by the stadiometer.

First all individuals were identified with more than 2 cm difference between consecutive height measurements and presumed that either the higher or the lower value is potentially invalid. Then logarithmic regression was performed for all combinations with potentially invalid values. According to the adjusted R-square of the different models the most unfitting values were excluded from the height data of such individuals. Altogether 1249 height values of 1182 individuals were excluded from the 102642 records.

We tested for obviously invalid haemodynamic parameters not compatible with life (pulse (P), systolic (SBP) and diastolic blood pressure (DBP)) in our dataset. Pulse: accepted between 40–200 beat/min (rejected:6 records), SBP: accepted between 70–220 Hgmm (rejected:75 records) and DBP: accepted between 30–120 Hgmm (rejected:46 records).

Running speed: accepted between 0.5–6.0 m/sec (rejected: 212 records).

### BMI and WHO z-score calculation

**Fig. 2:**
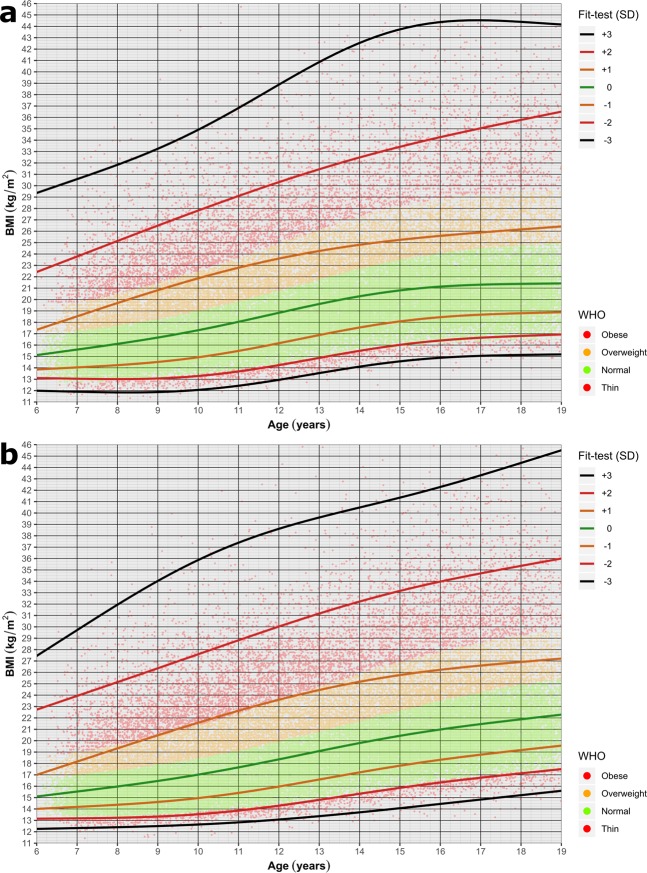
Body Mass Index (BMI) for Age plot. The lines represent SD (+3 to −3) values for BMI calculated for the whole tested population, for girls (**a**) and boys (**b**). Each individual is represented by a dot colour-coded based on the WHO criteria. Using the WHO criteria (−2 to +1 SD) we identified the normal-weight subpopulation (denoted as green points).

The BMI was calculated by the formula of $BMI=\frac{weight}{heigh{t}^{2}}$. The WHO z-scores were calculated by the methodology described in^28^. We used the WHO age and sex normalized LMS reference tables (https://www.who.int/growthref/who2007_bmi_for_age/en/). The z-score weight categories were determined according to the rules set by WHO (z-score < −3 - severely thin, −3 < = z-score < −2 - thin, −2 < = z-score < 1 - normal, 1 < = z-score < 2 - overweight, 2 < = z-score - obese). We also calculated the standard deviation (in range of −3 to +3) of experimental BMI values in our dataset for all age and sex categories. To compare the Hungarian population with the WHO data we visualized our data by colouring the individuals according to their WHO z-score weight categories and plotting the −3 to +3 standard deviation regression curves of the experimental BMI values by ggplot2 (Fig. 2).

### Generation of normal population hemodynamic dataset

Since obesity and being overweight influences cardiac parameters we included only the normal-weight subpopulation to generate a representative exercise-induced cardiac parameter dataset^29^. The WHO criteria (−2 to +1 SD z-scores) were used to identify the normal weight population (denoted as green points in Fig. 2). The running speed was calculated using the running time and distance values of the dataset.

From this normal subpopulation dataset, we also excluded the outlier measurements of the hemodynamic (before/after exercise pulse, SBP and DBP) and running speed based on the 1.5 IQR method^30^_._

As the running distance was selected by the class of the participant (1000 m for class 1–4 and 2000 m for class 5–8) and not by their age, we had some measurements of age >10 individuals with a 1000 m running distance and age <10 years with a 2000 m running distance. However, since the number of data points was greatly smaller than that of the other categories, we also excluded these measurement points from this dataset.

## Data Records

The dataset has been fully uploaded to the network, and users can download them through the figshare repository with the title Data Records^31^. The dataset comprises 2 data folders with 8 XLSX and 2 tsv files.

### Anthropometric dataset of 6–18-year-old children

The ***fit_database_anthropometric_all***.***xlsx*** is deposited in the anthropometric_all folder. Each data record contains the individual ID (that links different time series measurement dates of the same individual), measurement date, age (in years), age bin (age category in years), gender, height (cm), weight (kg) values, the calculated BMI, WHO z-score and WHO z-score categories of 102642 data acquisition points from 14267 individuals (7239 boys, and 7028 girls). We also provided this dataset as ***fit_database_anthropometric_all***.***tsv***.

The descriptive statistic (N, mean, SD) of this dataset for the different age and gender categories can be viewed in the XLSX table: ***descriptive_anthropometric_all***.***xlsx***.

The calculated gender- and age-specific height, body weight and BMI percentiles (1, 3, 5, 10, 25, 50, 75, 90, 95, 97, 99) and the corresponding number of individuals in this dataset can be found at the XLSX table: ***percentiles_anthropometric_all***.***xlsx***.

### Exercise induced cardiac parameter dataset

Each data record contains the individual **ID** (that links different time series measurement points of the same individual), **measurement date**, **age** (in years), **age bin** (age category in years), **gender**, **running distance**, **running speed** and the **0**′, **1**′, **5**′ and **10**′ **pulse**, **systolic blood pressure** (SBP), **diastolic blood pressure** (DBP) values collected from normal weight individuals according to the Fit-test protocol (Fig. 1) This dataset consists of 65345 data points of 10894 individuals (5408 boys and 5486 girls) and is deposited in the **excercise_normal** folder as ***fit_database_exercise_normal***.***xlsx*** (and as ***tsv*** file as well).

The descriptive statistic (N, mean, SD) of this dataset for the different age and gender categories can be viewed in the XLSX table: ***descriptive_ excercise_normal***.***xlsx***.

The calculated gender- and age-specific running speed and cardiac (pulse, systolic and diastolic blood pressures at 0′, 1′, 5′ and 10′ measurement points) for the normal weight Fit-test population can be found at the ***percentiles_runningspeed_exercise_normal***.***xlsx*** and the ***percentiles_cardiac_exercise_normal***.***xlsx*** tables.

The number of excluded outliers for each exercise-induced measurement (pulse, SBP, DBP and running speed are included in the **outlier_counts_exercise_normal**.**xlsx**.

## Technical Validation

### Measuring blood pressure

During the 12 years we used three different, ESH validated (https://www.healthcare.omron.co.jp/english/validation/europe.html) devices for measuring blood pressure (Omron3, Omron2, and URight TD3128). OMRON M3 blood pressure(BP) monitors are equivalent with the OMRON M6 BP device, which is validated (http://www.dableducational.org/) for children, obese and elderly^32–34^ and URight TD3128 Blood Pressure Monitor is also (ESH validation equal to TD3124, CE and FDA validated^35^.

The appropriate size cuff (small (17.0–22.0 cm), standard (22.0–32.0 cm) or large (32.0–42.0 cm) cuffs of OMRON devices, and 24–43 cm of TD-3128 devices) to the size of the child’s upper arm was used. If a cuff was too small, the next largest cuff was used even if it appeared larger than recommended^36^.

## Usage Notes

In the published datasets we provide repeated measurements on the same individuals of various anthropometric and hemodynamic parameters (102 642 records) of a large (14 267 participants) school-aged (6–18 years of age) cohort. It is prospective over 8 years (3.44 (SD 2.92) years, and 7.19 (SD 5.21) datapoints of participants).The anthropometric dataset can be used to analyze age and sex-dependent BMI changes leading to either obesity or normal body weight to identify risk-groups and proper time of intervention.

**Fig. 3:**
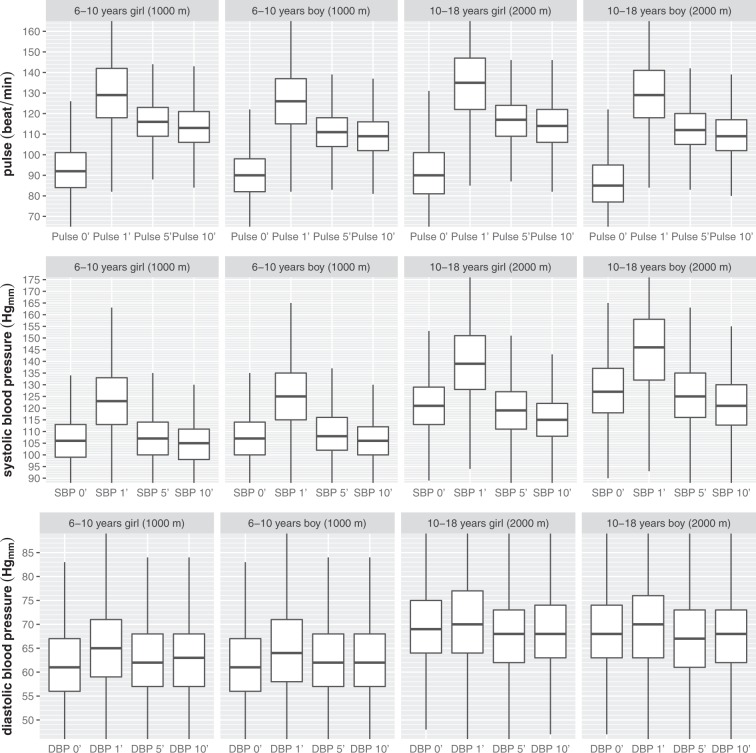
Pre and post Fit-Test hemodynamic data of the normal-weight subpopulation. Pulse, systolic (SBP) and diastolic blood pressure (DBP) data organised based on the age groups/running distance (6–10 and 10–18 years) and on the gender.

Fit-test allowed us to monitor the changes in the cardiovascular parameters before and after the DRT in normal-weight (age and gender separated) reference children and young adolescent (6–18 years of age) population (Fig. 3). The normal weight cardiac parameter dataset can be used as a standardized reference chart, to develop complex strategies utilizing exercise-induced parameters to screen for cardiovascular abnormalities.

The datasets are distributed in the normal standard file formats (text, xlsx) and can be read and processed by a variety of commonly used statistical packages, including SPSS, Matlab, Python, and R.
